# Translating and culturally adapting the shortened version of the Hospital Ethical Climate Survey (HECS-S) – retaining or modifying validated instruments

**DOI:** 10.1186/s12910-018-0274-5

**Published:** 2018-05-10

**Authors:** Pernilla Pergert, Cecilia Bartholdson, Marika Wenemark, Kim Lützén, Margareta af Sandeberg

**Affiliations:** 1 Childhood Cancer Research Unit, Department of Women’s and Children’s Health, Karolinska Institutet, SE-171 77 Stockholm, Sweden; 2 Children’s and Women’s Healthcare, Karolinska University Hospital, SE-171 76 Stockholm, Sweden; 3 Division of Community Medicine, Department of Medicine and Health Sciences, Linköping University, SE-581 83 Linköping, Sweden; 4 Centre for Healthcare Development, Region Östergötland, SE-581 85 Linköping, Sweden

**Keywords:** Cultural adaptation, Hospital ethical climate survey, Questionnaire, Translation, Trustworthiness

## Abstract

**Background:**

The Hospital Ethical Climate Survey (HECS) was developed in the USA and later shortened (HECS-S). HECS has previously been translated into Swedish and the aim of this study was to describe a process of translating and culturally adapting HECS-S and to develop a Swedish multi-professional version, relevant for paediatrics. Another aim was to describe decisions about retaining versus modifying the questionnaire in order to keep the Swedish version as close as possible to the original while achieving a good functional level and trustworthiness.

**Methods:**

In HECS-S, the respondents are asked to indicate the veracity of statements. In HECS and HECS-S the labels of the scale range from ‘almost never true’ to ‘almost always true’; while the Swedish HECS labels range from ‘never’ to ‘always’. The procedure of translating and culturally adapting the Swedish version followed the scientific structure of guidelines. Three focus group interviews and three cognitive interviews were conducted with healthcare professionals. Furthermore, descriptive data were used from a previous study with healthcare professionals (*n* = 89), employing a modified Swedish HECS. Decisions on retaining or modifying items were made in a review group.

**Results:**

The Swedish HECS-S consists of 21 items including all 14 items from HECS-S and items added to develop a multi-professional version, relevant for paediatrics. The descriptive data showed that few respondents selected ‘never’ and ‘always’. To obtain a more even distribution of responses and keep Swedish HECS-S close to HECS-S, the original labels were retained. Linguistic adjustments were made to retain the intended meaning of the original items. The word ‘respect’ was used in HECS-S with two different meanings and was replaced in one of these because participants were concerned that respecting patients’ wishes implied always complying with them.

**Conclusions:**

The process of developing a Swedish HECS-S included decisions on whether to retain or modify. Only minor adjustments were needed to achieve a good functional level and trustworthiness although some items needed to be added. Adjustments made could be used to also improve the English HECS-S. The results shed further light on the need to continuously evaluate even validated instruments and adapt them before use.

## Background

### The ethical climate

The organizational climate is often considered as the organization’s personality and how the organization is perceived by group members [1]. The organizational climate includes the ethical climate, that is the individual healthcare professionals’ perceptions of the organization’s atmosphere, which influences behaviours and attitudes [2]. According to Spencer and Mills [3], the ethical climate “defines the organization in both its internal and external relationships and permeates the whole” ([3], p. 325). The ethical climate in the context of healthcare has been defined as the “implicit and explicit values that drive healthcare delivery and shape the workplaces in which care is delivered” ([4], p.24). Furthermore, Silen et al. [5] highlight the interconnection between a positive ethical climate and good care. How well ethical issues are handled is influenced by the workplace’s ethical climate, and a non-supportive ethical climate contributes to moral stress [6]. A positive ethical climate is characterised by a shared vision of care and teamwork where members inform and support each other, and promotes the ability to meet the needs of patients and their families [5]. A positive correlation has been demonstrated between ethical climate and nurses’ job satisfaction and ultimately the quality of patient care [7, 8]. Also, when studying nurses’ perceptions of the overall ethical climate, Abou Hashish [9] has shown a positive correlation with job satisfaction and a significant negative correlation with turnover intention. It is important that healthcare organizations evaluate the ethical climate from a multi-professional perspective, and promote a supportive working environment [10] that enhances patient care [8].

### Measuring the ethical climate

To evaluate the ethical climate, one needs a validated instrument appropriate to the setting in question. Based on a questionnaire by Cullen et al. [11], the Hospital Ethical Climate Survey (HECS) was developed in the USA by Olson [12] and found to have good psychometric properties (Cronbach’s alpha: 0.91) [2]. HECS has the following dimensions: nurses’ relationships with peers, patients, managers, physicians and the hospital [2]. HECS is an instrument for assessing nurses’ perceptions of the ethical climate at their workplace. Some of the items concern support in deciding what is right and wrong, although most items are quite general but intend to capture the values that shape the ethical climate of the workplace [6]. HECS has been translated into Swedish and tested in a pilot study (Cronbach’s alpha: 0.85) [6]. A shortened version of the English HECS (HECS-S) has been developed more recently [13] and found to have good internal consistency for physicians (Cronbach’s alpha: 0.77) and nurses (Cronbach’s alpha: 0.87). Thus, all the above-mentioned versions of the questionnaire claim good validity and reliability.

It is important that validated instruments are used to ensure that measurements have good validity and reliability. However, even validated instruments need to be adapted before use in new contexts; and also need to be re-evaluated continuously because language and culture are dynamic and change over time [14]. When evaluating a questionnaire, it is important to focus not only on the content, but also on the instrument’s functional level to facilitate the respondents’ cognitive process of understanding and answering the items [15]. Translating and adapting an instrument to a new context therefore often poses the dilemma of whether to retain an identical content or modify and adapt to the new context.

To ensure a Swedish version as close as possible to the most recently developed HECS-S, and thus one with comparable results, it was considered to be an advantage to start from the HECS-S, even though there was already a Swedish HECS. Furthermore, HECS-S is directed not only at nurses but also at physicians. All the professionals in the healthcare team affect the ethical climate at the workplace and thus a multi-professional questionnaire is needed. Furthermore, in a paediatric context, the questionnaire also needs to acknowledge the central role of parents.

The aim of this study was to describe the process of translating and culturally adapting HECS-S and also to develop a Swedish, multi-professional version, relevant to paediatrics. Another aim was to describe decisions about retaining versus modifying the questionnaire, an important part of the process, in order to keep the Swedish version as close as possible to the original version and still achieve a good functional level and trustworthiness.

## Methods

In this study, HECS-S was translated and culturally adapted to the Swedish context, using and evaluating the existing Swedish translation of the original HECS [6]. Data from a previous study using a modified version of the Swedish HECS, were also utilized in the evaluation [16].

### The Hospital Ethical Climate Survey (HECS)

The original HECS consisted of 26 items phrased as statements concerning nurses’ perceptions of the ethical climate at their workplace. On a 1–5 Likert scale, the respondents were asked to indicate the veracity (almost never true, seldom true, sometimes true, often true and almost always true) of each statement (Table 1). In the Swedish HECS [6], the respondents were asked to indicate on each statement how often (never, almost never, sometimes, almost always and always) the situation corresponded to their experiences and perceptions of the situation (Table 1). A modified Swedish HECS has previously been used in the paediatric oncology context using the same scale as the Swedish HECS [16]. In that study, the same questionnaire was used for physicians, registered nurses and nursing assistants. In the modified Swedish HECS, thirteen items were excluded, including six items regarding support from the manager. Furthermore, four items were added, including: three items concerning the relationships between nurses and nursing assistants, and one item concerning parents’ wishes, relevant to the paediatric setting (Table 1). The three items concerning the relationships between nurses and nursing assistants were formulated as existing items about the relationships between physicians and nurses. The shortened version of HECS (HECS-S) includes 14 items and has different versions for nurses and physicians, respectively [13]. Just as in the original version, the respondents are asked to indicate the veracity of the statement in relation to the reality on their unit, ranging from 1 to 5 (1 = almost never true, 5 = almost always true) but now with verbal labels only at the two end-points (Table 1).

**Table 1:** Overview of differences between available versions of HECS

	Targeted population			Number of items	Labels of the scales
	Nurses	Physicians	Nursing ^a^		
Versions of HECS					
Original HECS	X			26	Almost never true, Seldom true, Sometimes true, Often true, Almost always true
Swedish HECS	X			26	Never, Almost never, Sometimes, Almost always, Always
Modified Swedish HECS	X	X	X	13 + 4 new	Never, Almost never, Sometimes, Almost always, Always
HECS-S	X	X		14	Almost never true – Almost always true

^a^ Nursing assistants

In a previously published study [16], the Swedish HECS was used after permission had been obtained from Lützén. In this previous study, healthcare professionals (physicians, nurses and nursing assistants) working at three different paediatric units, were invited to answer the modified Swedish HECS. A total of 89 questionnaires were completed including responses from: physicians (*n* = 15), nurses (*n* = 36) and nursing assistants (*n* = 38). Data from this previous study were used in the present study and data analysis was performed using the Statistical Package for Social Science (SPSS). To illustrate the distribution of answers, frequencies of selected Likert scale alternatives were calculated by pooling answers to all items [17].

### Procedures of translating and culturally adapting HECS-S

The overall procedure follows the scientific structure of two rigorous guidelines [18, 19] and includes the following steps: preparations, translations, synthesis of the translated versions, cognitive debriefing, review of cognitive debriefing, back translation and review of back translation.

#### Preparations [19]

Dr. Olson, the original designer of HECS, and Dr. Hamric, who shortened the original version, were contacted and permission was obtained to translate and culturally adapt HECS-S so as to develop a Swedish version.

#### Translations [18]

The translation of HECS-S from English into Swedish was done by the first (PP) and last (MafS) authors, both with a good knowledge of the paediatric context and ethics, and highly proficient in written as well as spoken Swedish and English.

#### Synthesis of the different versions [18]

The translated items in HECS-S were compared to the corresponding items in the Swedish HECS [6] as well as in the modified Swedish HECS [16]. The synthesis of the different versions was performed by a review group (*n* = 6) with expertise in ethics, paediatrics and linguistics, including the first, second, fourth and fifth authors. The comparisons were performed in order to achieve consensus and synthesize the versions into one. Concepts and the different translations of the words were discussed. Furthermore, the discussion concerned which items to include in the Swedish HECS-S, and how to make sure that all professional groups were included. Each issue discussed and the final solution were documented in written notes.

#### Cognitive debriefing [19]

Face validity, content validity and respondent satisfaction were tested through focus group interviews and cognitive interviews with the target population. Three focus group interviews were conducted by the first (PP) and last (MafS) authors with healthcare professionals, including: two Swedish national networks of consultant nurses (*n* = 14), a working group on ethics including paediatric oncology nurses and paediatric oncologists (*n* = 8) and experienced paediatric nurses (n = 8). In the focus group interviews, all the participants had the questionnaire and discussed it one item at a time. The moderator’s role was to encourage participants to reflect on the relevance of the items, how it could be understood and whether any items were missing. Notes were written on the questionnaire by the moderator, and some participants made notes in their questionnaire during the focus group. Three cognitive interviews [20] were performed by the third author (MW) with healthcare professionals in paediatric cancer care, including: RNs (*n* = 2) and a nursing assistant (*n* = 1). To explore how the participants understood the items and why they answered as they did, they were encouraged to “think aloud” when answering the questionnaire. The interviewer took notes and interfered as little as possible. After the questionnaire had been completed, a few follow-up questions were asked about things the interviewer had noticed during the “think-aloud” phase, which led to brief discussions about, for example, ambiguous terms.

#### Review of cognitive debriefing [19]

Decisions on whether to retain or modify, based on data from the focus groups and cognitive interviews, were discussed in the review group (all authors), including the third author (MW) with an expertise in questionnaire design. For example, new relevant items were suggested during focus group interviews and were added after in-depth discussions in the review group.

#### Back translation [18]

The preliminary Swedish version of HECS-S was translated back into English by a native speaker and certified translator. This translator was uninformed, meaning that he had no knowledge of the context, and was seeing the questionnaire for the first time. Thus, the back translation was made so as to ensure equivalence of the item content with the original English versions.

#### Review of the back translation [19]

The review group compared the wordings of the relevant items in the original English versions (HECS-S and HECS) and the back-translated version. Discussions in the review group did not lead to any further revisions of the Swedish HECS-S.

## Results

The translation and cultural adaptation of HECS-S aimed to achieve a conceptual equivalence with the original questionnaire, but also to produce a Swedish version with a good functional level and trustworthiness. The questionnaire had a relatively good functional level in that participants in focus groups and cognitive interviews mostly found it easy to understand and answer, and only minor adjustments were made to questionnaire content, design, language, and to cultural matters in the Swedish version.

### Adjustments to the content of the questionnaire

The Swedish HECS-S consists of a total of 21 items including: all 14 items from the HECS-S; one reintroduced item from the original HECS and the Swedish HECS; four items from the modified Swedish HECS; and two completely new items (Table 2).

**Table 2:** Items included in the Swedish HECS-S in addition to the 14 items from the HECS-S

Origin of the items	Added items
Original HECS / Swedish HECS	Physicians ask registered nurses (RN) about their opinion regarding treatment decisions.
Modified Swedish HECS	RN and nursing assistants (NA) trust one another. RN and NA respect each other’s opinions even when they disagree about what is best for the patients. RN ask NA about their opinions regarding care decisions.
	The parents’ wishes are considered.
New items	Ethical issues are identified on my unit On my unit we talk about different ways of dealing with ethical issues

The reintroduced item from the original HECS/Swedish HECS, concerning physicians asking nurses their opinions on treatment decisions, had been deleted in the HECS-S and was reintroduced in the Swedish HECS-S (Table 2). This item was also one of the items complemented with an additional item concerning nurses asking nursing assistants for their opinions on care decisions from the modified Swedish HECS (Table 2).

Nursing assistants were added as a target group for the questionnaire, and some items were adapted or modified in order to include them, in accordance to the modified Swedish HECS (Table 2). Furthermore, the original HECS and HECS-S both include a statement regarding the patient’s wishes being respected. Even though, in the paediatric setting, the patient is a child, it was decided not to specify this. In the modified Swedish HECS, an item was added concerning parents’ wishes, and this statement was also added to the Swedish HECS-S (Table 2). These four items, concerning nursing assistants and parents, can easily be removed for use in settings where they would not be relevant.

Cognitive interviews revealed that items explicitly asking about identifying and dealing with ethical issues were missing. Thus, two completely new items were added (Table 2) and were formulated as follows: “Ethical issues are identified on my unit” and “On my unit we talk about different ways of dealing with ethical issues”. In an attempt to keep the Swedish version as close as possible to HECS-S and avoid context effects, these two statements were added at the end of the questionnaire.

### Adjustments to questionnaire design

Adjustments to the design were made to further increase the questionnaire’s functional level and facilitate data collection. Both the original HECS and HECS-S have a long introductory text about how the respondents should think while responding; to increase the chances that respondents would both read and understand this text, it was shortened in accordance with the Swedish HECS, which begins with a brief definition of the concept “ethical climate” [6]. Thereafter, the instructions were kept in accordance with HECS-S to highlight the fact that participants who work at several units should think of the unit where they work most frequently [13].

**Fig. 1:**
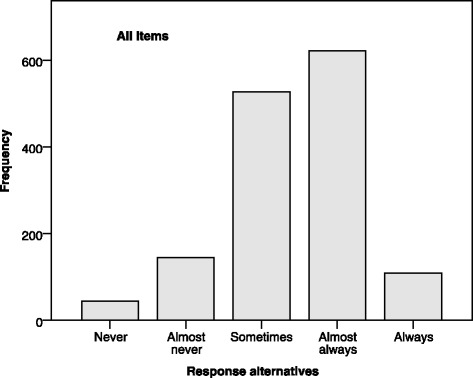
Frequencies of response alternatives selected by healthcare professionals (*n* = 89) on all items in the modified Swedish HECS, using data from Bartholdson et al. [16]

**Fig. 2:**
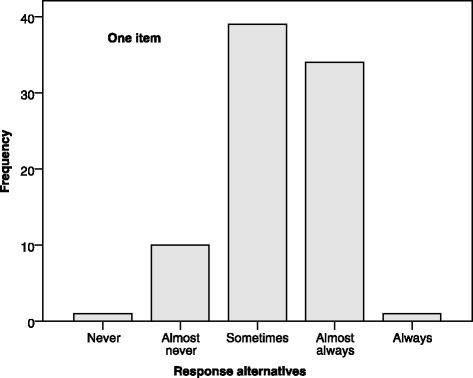
Frequencies of response alternatives selected on the item: “The atmosphere encourages us to ask questions, learn and seek creative solutions to ethical problems in patient care”, using data from the previous study [16]

In the previous study, using the modified Swedish HECS [16] with the scale ranging from never to always, the results showed that few respondents selected the scale’s extreme alternatives (Fig. 1). The mean score was 3.43 (SD = 0.39), with a range of 2.06–4.33. Furthermore, a core item has been chosen as an example to demonstrate the distribution of answers (Fig. 2).

Using never and always as the end-points of the scale led to a concentration of answers to the other three alternatives. Thus, in the Swedish HECS-S, the Likert scale’s labels “almost never–almost always” were chosen to stay close to the original scale; furthermore, only the extreme alternatives were specified, in accordance with HECS-S. This decision was in accordance with the aim of ensuring comparability with results from the English versions of the original HECS and HECS-S and possibly to achieve a more even distribution of responses.

To facilitate data collection, the ambition was to achieve a joint questionnaire that was relevant and easy for all healthcare professionals to answer. The different wording in HECS-S that distinguishes the versions for physicians and healthcare professionals, respectively, are the words for “supervisory physician” and “manager”. In the Swedish healthcare organization, a correct expression is the immediate (Swedish word: *närmaste*) manager, thus this could be used for all healthcare professionals. In addition, in HECS-S the term “medicine” was used for physicians and “nursing” was used for nurses, and these were changed to the Swedish word for “healthcare” or “care” which in the Swedish context includes both medical and nursing care and thus could be used for all healthcare professionals.

It could be argued that two of the statements incorporate several issues: “There is a sense of questioning, learning, and seeking creative responses…” and “The feelings and values of all parties involved … are taken into account …”. However, respondents did not experience difficulties in answering these statements, and it was decided that these items are not about the individual words but rather try to capture a common experience/sense exemplified and explained by the contents.

### Linguistic adjustments

Linguistic adjustments to the translation into Swedish were made in order to retain as much as possible of the original items’ intended meanings. Instead of using the exact translation of a concept, a word that would naturally be used in the Swedish context was chosen. For example, in cognitive interviews participants were concerned as to whether respecting patients’ and parents’ wishes also implied implementing unrealistic wishes about treatment in palliative care. Thus, to capture the conceptual meaning in the healthcare setting, “respected” was replaced with the Swedish word for “taken into account” (*beaktas*) and the statements were accordingly formulated as follows: “The patients’ wishes are taken into account” and “The parents’ wishes are taken into account”. In all former versions of HECS, the same word “respect” is also used about the relationships between different professional groups, but for these items the respondents in the interviews did not express any doubts about the meaning of the word.

One statement in the original HECS and HECS-S is about “a sense of questioning, learning, and seeking creative responses” which in the Swedish HECS was translated as “an atmosphere that encourages questioning…” (Swedish words: *en stämning som uppmuntrar*). However, because, in the focus group interview, this was regarded as an indistinct formulation, it was reformulated as follows: “There is an openness to asking questions, learning and seeking creative solutions…”.

### Cultural adjustments to the Swedish context

Cultural adjustments to the Swedish context were made so as to ensure that items were relevant to the structure and organisation of Swedish society and healthcare. The terms “peers” and “colleagues” are used in HECS-S, but in the Swedish HECS-S the term “co-workers” was chosen to ensure that the items would be relevant and include all professional groups.

One item is about the “hospital’s mission” (Swedish word *uppdrag*) and in the cognitive interviews this raised questions about whether it referred to health professionals’ mission to make people healthy or to a mission assigned by, for example, politicians. The Swedish HECS translated mission as “care ideology” but this could vary between different wards at a hospital. Thus, mission was re-worded as “hospital goals/values” to clarify what was intended in the Swedish context and better capture the conceptual meaning.

## Discussion

In this study, HECS-S has been translated and culturally adapted, and a Swedish, multi-professional version has been developed, relevant to the paediatric context. In the process, the aims were to keep the Swedish version as close as possible to the original version while achieving a good functional level and trustworthiness. Based on focus groups and cognitive interviews, the questionnaire showed a relatively good functional level; only minor adjustments were needed. Translating and adapting a validated instrument to a new context often involves a dilemma of whether to retain an identical content or to modify and adapt it to the new context. The possibility of comparing results from different studies and contexts is an argument for retaining as much of the instrument as possible. However, maintaining the content’s validity between cultures calls for cultural adaptation [18, 21]. Furthermore, adapting to a new context and continuously improving the instruments’ functional level are arguments for making modifications to achieve face validity and trustworthiness both as regards respondents and the recipients of the results.

The process of translating and culturally adapting was performed slightly differently than usual because it had already been undertaken by Lutzen et al. [6] with the original HECS; thus the Swedish HECS could be used for the comparison with the new translation. Furthermore, the back translation was performed late in the process and did not lead to any new modifications of the Swedish HECS-S. Schuster et al. [22] have argued that a back translation by an uninformed native speaker of the original language is not necessary when the authors have excellent skills in the original and target languages. Furthermore, if many concepts are contextual, as in HECS-S, it could even be seen as counter-productive to use an uninformed translator.

The original HECS intends to capture perceptions of the ethical climate [2] which are the “implicit and explicit values” that form the workplace ([4], p.24). Most statements are quite general and concern support in dealing with patient care situations/problems, and one could argue that this rather captures psychosocial support and the psychosocial climate. It is likely that these concepts overlap, because HECS-S also includes items concerning support in decisions on right and wrong. Furthermore, in this study more specific items about identifying and actually dealing with ethical issues were added. Participants found that items covering these issues were missing, based on their perception of what captures the ethical climate in their clinical practice at their workplace. The intention of the interviews was not to explore the underlying values or the concept of ethical climate but to ensure the relevance of the present items and identify potentially lacking issues.

Good validity and reliability have been reported for the original HECS [2] and HECS-S [13], as well as for the Swedish HECS [6], despite differences in the number of items and the scale’s labels. In the Swedish HECS-S, it was decided to keep the scale’s original labels, in accordance with the aim of keeping the Swedish HECS-S as close to the original as possible. It could be a drawback that respondents who might have chosen the extreme alternatives – ‘never’ or ‘always’ – no longer have that opportunity. However, when the scale ranged from ‘never’ to ‘always’, the results showed that just a few respondents selected the extreme alternatives, implying that in practice the scale had only three alternatives. Thus, avoiding these extreme alternatives could potentially lead to a more even distribution of responses. To illustrate this, data from the previous study, using the modified Swedish HECS, were used to calculate the mean score 3.43 (SD = 0.39), and range 2.06–4.33, which could be compared with a study by Pauly et al. [23] using the original HECS with a mean score of 3.48 (SD = 0.612), and a range of 1.73–4.96. There is an indication of a wider range, i.e. less concentration of answers in the middle of the scale, in the study using the original HECS scale. Calculations of mean scores and SD using ordinal variables can be and have been questioned [24]. However, this was the only possibility of making a comparison with published studies using the original HECS scale, as most publications found use these methods.

The original HECS was designed for nurses and the HECS-S for nurses and physicians. The aim with the Swedish HECS-S was to reach all major professional groups working in direct patient care in the Swedish healthcare system, and therefore nursing assistants were included. Swedish nursing assistants, with a role similar to that of a Licensed Practical/Vocational Nurse in the American healthcare organisation, play an important role in the healthcare team [25]. Furthermore, the ambition to achieve a single questionnaire to facilitate data collection appropriate for all these groups, could potentially lead to some items not being relevant for some. For example, the three added items regarding the relationships between nurses and nursing assistants could be considered as not relevant for physicians. However, respondents are asked, on all items, to base their answers on their perceptions, and relationships between nurses and nursing assistants are very relevant also for physicians’ ethical climate because they all belong to the same team. The endeavour to make the Swedish HECS-S relevant for as many as possible resulted in more items and thus it could be argued that the instrument is no longer a short version. However, the added items are easily removed if they are not relevant.

In a qualitative study, nurses mentioned “complying with patients’ wishes” as one aspect of promoting a positive ethical climate [5]. Participants in the present study were confused about whether respecting patients’ wishes meant taking them into account or always complying with them, even unrealistic wishes about treatment. Thus, in the item concerning the patient’s wishes and the added item concerning the parent’s wishes, the word “respected” was changed to “taken into account” in the Swedish HECS-S. If the word “respect” in relation to a wish could be interpreted differently by respondents, it would make it difficult to understand the results on this item. Since “taken into account” was chosen for the Swedish HECS-S, caution is required when analysing future data from this item and when comparing it with other studies using HECS-S. Beach et al. [26] have argued that the word respect is frequently used in ethics and healthcare without a common definition, and they questioned whether respect for a person is the same as respect for autonomy or a patient’s wish. Thus, one could argue that it is unfortunate to use the same word for these different meanings in a questionnaire. Furthermore, the results of the present study indicate that understanding and answering the items were easier when the object of the respect was a person/professional group rather than a wish. This reinforces our claim that even established questionnaires need to be continuously re-evaluated and revised, since the meaning of words may change over time and translation cannot focus exclusively on literal translation, but needs to capture the conceptual meaning. Furthermore, we would argue that the use of the word respect should be scrutinized and carefully considered also in HECS-S.

One could question why HECS-S includes just one item concerning the relationship with patients, since the interaction with patients constitutes a large part of daily professional healthcare work and it is, therefore, reasonable to assume that it influences the ethical climate. Additionally, to add an item regarding parents’ wishes in a paediatric context is hardly controversial because family-centred care predominates in paediatric care [27]. Thus, much of the communication is with the parents. It could, however, be argued that the item related to the parents’ wishes would also be highly relevant with regard to next-of-kin in adult/geriatric care. Next-of-kin was not included in the original HECS [2], but Tonnessen et al. [28] argue that the attitudes to next-of-kin need to be included in the concept of the workplace’s ethical climate. Additionally, the adaptation process to make the questionnaire relevant for use in paediatrics resulted in only one added item. This item could easily be made equally relevant for adult settings by changing only one word as suggested above. This indicates that a special paediatric version is not needed. Even though all interviews in the present study have been conducted in the paediatric setting, we would argue that the questionnaire is also relevant in other healthcare settings.

It is noteworthy that the adjustments made to the Swedish HESC-S including the added items could equally be used to improve the functional level of the English HECS-S. The results further shed light on the importance that even validated instruments with a good functional level need to be continuously evaluated and modified.

## Conclusions

In the process of translating and culturally adapting HECS-S so as to develop a Swedish version, the repeated decisions on whether to retain or modify the instrument were a major element. The results showed that only minor adjustments were needed in order to achieve a good functional level and trustworthiness for the Swedish HECS-S. However, in order to develop a multi-professional and paediatric version, and also to better capture the ethical climate, specific items needed to be added. In the present study, the focus has been on experts and healthcare professionals rather than statistical measures. However, further evaluations of the Swedish HECS-S are needed with modern test theory, such as RASCH.

## Abbreviations

HECS: Hospital ethical climate survey
HECS-S: Hospital ethical climate survey-short
